# Cutaneous metastasis of ascending colon cancer harboring a BRAF V600E mutation: A rare case report

**DOI:** 10.1097/MD.0000000000020026

**Published:** 2020-05-22

**Authors:** Lianggong Liao, Qian Cheng, Guangsheng Zhu, Feng Pei, Shengwei Ye

**Affiliations:** a Department of Gastrointestinal Surgery, Hubei Cancer Hospital, Tongji Medical College, University of Science and Technology, Huazhong; b Colorectal Cancer Clinical Research Center of Wuhan; c Colorectal Cancer Clinical Research Center of Hubei Province, 116 Zuodaoquan South Road, Wuhan, Hubei, China.

**Keywords:** ascending colon cancer, case report, cetuximab, cutaneous metastasis, FOLFIRI, vemurafenib

## Abstract

**Rationale:**

Cutaneous metastases from colorectal cancer are extremely rare and generally appear several years after diagnosis or resection of the primary colorectal tumor. Although cutaneous metastasis is unusual, it often indicates a poor prognosis.

**Patient concerns:**

We treated a 62-year-old woman with multiple cutaneous metastatic nodules on the chest, back, and armpit 7 months after resection of ascending colon cancer.

**Diagnoses:**

The patient was diagnosed with cutaneous metastasis of ascending colon cancer with BRAF V600E mutation.

**Interventions:**

After 6 cycles of fluorouracil, leucovorin, oxaliplatin, cetuximab, and emurafenib, most of the metastatic lesions had begun to shrink, and no new metastases were observed. Serum tests showed that the levels of several tumor markers were decreased.

**Outcomes:**

The patient responded well to treatment and survived for 6.5 months after presentation with skin metastasis.

**Lessons:**

Cutaneous metastasis of colorectal cancer with BRAF V600E mutation is a rare but important phenomenon that should not be ignored. Cutaneous metastasis of colorectal cancer frequently indicates advanced disease and poor prognosis. The SWOG 1406 program is one of the treatment options, but this needs further exploration.

## 1 Introduction

Metastasis is a key factor that affects the prognosis of cancer patients. Breast cancer in women and lung cancer in men often involve skin metastasis,^[1]^ although the incidence of cutaneous metastasis from a primary visceral tumor is only about 0.7% to 9%.^[3]^ Colorectal cancer commonly metastasizes to the regional lymph nodes, lung, liver, and peritoneum but rarely to the cutaneous tissue.^[2]^ The incidence of cutaneous metastases of colorectal cancer is less than 4%,^[4]^ and it indicates a poor outcome when it is a disseminated disease. The mean survival time for patients with soft-tissue metastases is 5.4 months.^[5]^

Here, we report a rare case of multiple cutaneous metastatic nodules at the chest, back, and armpit after curative resection and adjuvant chemotherapy for ascending colon cancer. The patient was treated to help with optimal clinical evaluation and for cutaneous metastasis of colorectal cancer. The patient provided informed consent for publication of the case. This case report was approved by the Ethics Committee of Hubei Cancer Hospital.

## 2 Case report

A 62-year-old Chinese woman was diagnosed with right colon cancer (Stage III) and underwent radical surgery. The histopathological report revealed a moderately di-erentiated adenocarcinoma infiltrating the serosal layer with lymph node involvement. The pathologic stage was pT4N1M0 according to the American Joint Committee on Cancer 8th edition. The patient received 6 cycles of adjuvant chemotherapy with fluorouracil, leucovorin, and oxaliplatin.

**Figure 1:**
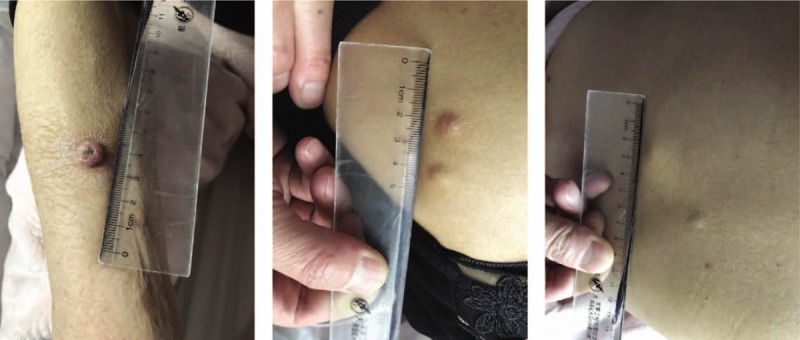
Cutaneous nodules on the skin.

Seven months after surgery, the patient presented with multiple cutaneous metastatic nodules at the chest, back, and armpit. Approximately 3 months after chemotherapy, cutaneous masses 2 to 5 cm in diameter that were rubbery hard, well-demarcated, and mildly tender were observed at the chest, back, and armpit (Fig. 1). The left supraclavicular and inguinal lymph nodes could not be palpated. The levels of serum tumor markers were elevated, including carcinoembryonic antigen (CEA,179.7 μg/L) and carbohydrate antigen 19–9 (36.83 U/ml).

**Figure 2:**
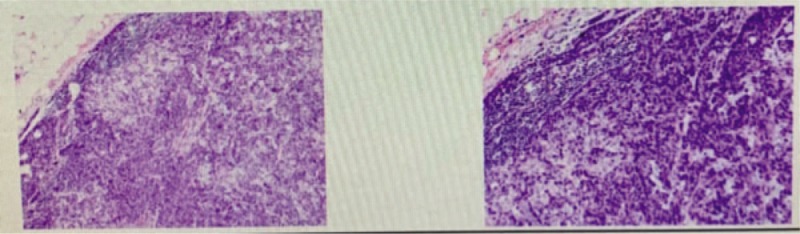
Biopsy from a nodule showing metastatic adenocarcinoma. (H&E; 400 × ).

The patient underwent a cutaneous mass biopsy, and the histopathology showed a malignant neoplasm composed of well-formed ductal structures that had infiltrated the dermis. The epithelium was composed of columnar cells of pleomorphic vesicular nuclei with more than 1 nucleolus and frequent atypical mitotic figures. The stroma presented a mixed inflammatory reaction of lymphocytes, eosinophils, neutrophils, fibrosis, and ectatic vessels. The findings were compatible with metastatic adenocarcinoma (Fig. 2). Immunohistochemistry indicated that the lesions were positive for cytokeratin (CK) 20, CDX2, and SATB2 and negative for CK7, confirming the colorectal origin. The PD-1 positive percentage was <5%, and the PD-L1 percentage was <5%. KRAS, NRAS, and PIK3CA were all wild-type, although the tumor was found to harbor the BRAF V600E mutation.

**Figure 3:**
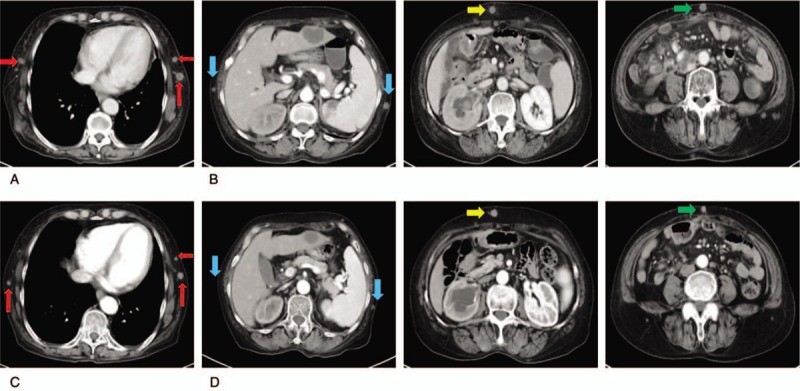
CT indicating cutaneous lesions on the chest wall (A) and abdomen wall (B). (C) Chest CT and abdominal CT (D) after 2 cycles of FOLFIRI and cetuximab treatment. CT = computed tomography. FOLFIRI = irinotecan, leucovorin, and fluorouracil.

Chest computed tomography (CT) indicated multiple metastases in the chest wall and back (Fig. 3A). Abdominal CT showed multiple enhanced nodules located in the abdomen, pelvic wall, and the muscle spaces, with a longest diameter of about 2.0 cm (Fig. 3B).

**Figure 4:**
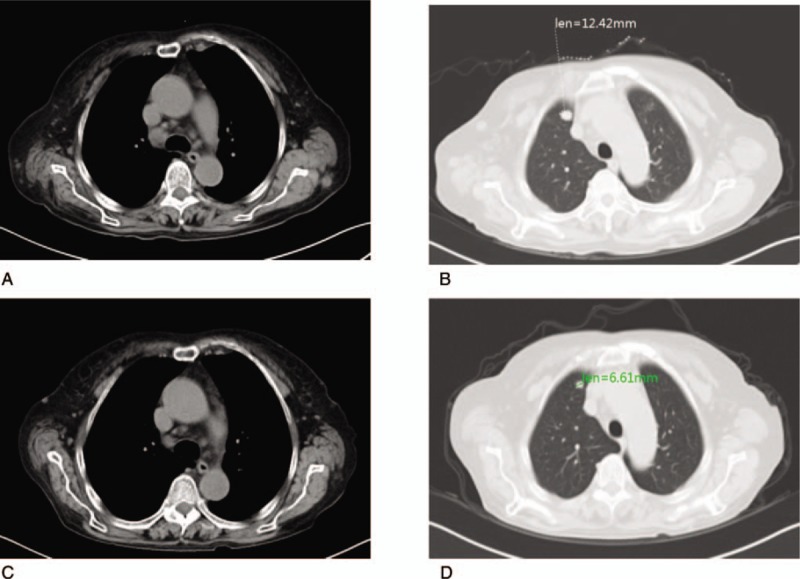
Lymph nodes in the mediastinum (A) and metastatic nodules in the right upper lung (B) before and after FOLFIRI and cetuximab treatment (C and D). FOLFIRI = irinotecan, leucovorin, and fluorouracil.

Multiple nodules could be seen in the mediastinum, and the largest lesions short diameter was about 1.3 cm (Fig. 4A). There was a metastatic nodule in the right upper lung with a long diameter of about 1.2 cm (Fig. 4B).

**Figure 5:**
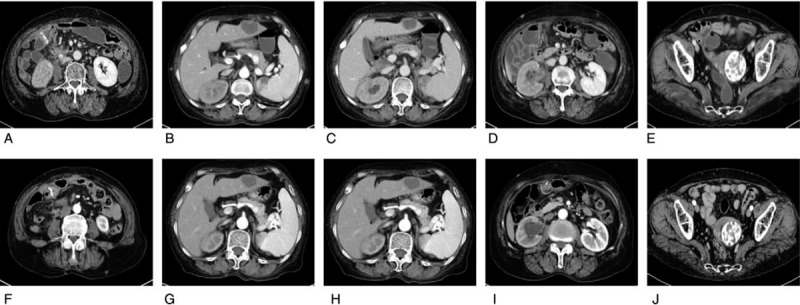
The right abdominal abdomen anastomotic stoma wall (A) and metastatic nodules in the third segment of the liver (B), spleen (C), the right renal pelvis subsided (D) and abdominal pelvic cavity (E) before and after FOLFIRI and cetuximab treatment (F to J). FOLFIRI: irinotecan, leucovorin, and fluorouracil.

The right abdominal anastomotic stoma wall was thickened and enhanced with mild ring enhancement; we considered this a recurrence (Fig. 5A). A low-density mass was seen in the third segment of the liver; the border was slightly blurred and mildly unevenly enhanced. The size of this mass was about 3.7 cm × 3.0 cm (Fig. 5B). A slightly flaky area in the spleen was also considered a metastasis (Fig. 5C). A slightly high-density mass with an unclear border was seen in the right renal pelvis, the right renal pelvis was slightly dilated, and the right ureter was unclear (Fig. 5D). Multiple masses were seen in the mesentery and retroperitoneal and abdominal pelvic cavities. The largest were about 6.2 × 3.1 cm with mild enhancement (Fig. 5E).

We administered FOLFIRI (irinotecan 240 mg, calcium leucovorin 0.2 g, fluorouracil 500 mg intravenous bolus, 5-FU 3000 mg pumped for 46 hours) in combination with the EGFR inhibitor cetuximab (700 mg) and the BRAF inhibitor vemurafenib (960 mg PO twice, d1-d10, q14d). After the second cycle of FOLFIRI and cetuximab, most of the metastatic lesions had begun to shrink, and no new metastases were observed. Serum tumor markers were decreased (CEA: 66.58 μg/L and carbohydrate antigen 19–9: 17.59 U/ml).

After 2 cycles (4 weeks), the metastatic nodules in the chest wall, abdomen, and pelvic wall had decreased in size (Fig. 3C and D). Multiple lymph nodes in the mediastinum had also shrunk after chemotherapy and cetuximab treatment (Fig. 4C and D).

The anastomotic wall was slightly thickened, and the area of slight enhancement showed no significant changes (Fig. 5F). The low-density mass in the third segment of the liver had a long diameter of about 3.1 cm, which was smaller than prior to treatment (Fig. 5G). The long diameter of the spleen metastatic nodule had decreased to 0.7 cm (Fig. 5H). The slightly higher density mass in the right renal pelvis had shrunk (Fig. 5I). There were still multiple masses in the mesentery and retroperitoneal and abdominal pelvic cavities. The largest were about 3.6 × 2.4 cm (Fig. 5J). The tumor response was evaluated as partial response according to the Response Evaluation Criteria In Solid Tumors after 2 cycles. She received a total of 6 cycles of FOLFIRI in combination with cetuximab with vemurafenib. According to the Common Terminology Criteria for Adverse Events Version 4.0, the patient developed grade 3 white blood cell decline, grade 2 vomiting reaction, and mild skin toxicity caused by cetuximab, which improved after symptomatic treatment. After 6 cycles of treatment, the patient died of disease progression.

## 3 Discussion

Metastatic skin cancer is defined as skin metastases from visceral cancer, excluding primary skin cancers, and hematological malignancies.^[6]^ Patterns of cutaneous metastases vary between women and men. Breast cancer, colorectal cancer, and melanoma frequently metastasize to the skin in women. In men, lung cancer, melanoma, and colorectal cancer are the most common cause of cutaneous metastases.^[8]^ Cutaneous metastasis from colorectal cancer is rare; the incidence ranges from 2.3% to 3.4%.^[7]^ The most frequent sites of cutaneous metastasis from colorectal cancer are the abdomen, extremities, perineum, head, neck, and penis. These metastases generally occur within the first 2 years after surgery and often present simultaneously with metastases to the liver, peritoneum, and lung.^[9]^ Cutaneous metastasis from an internal malignancy indicates poor prognosis. Survival after diagnosis ranges from 1 to 34 months.^[15]^ Therefore, skin metastasis from colorectal cancer suggests dissemination of the disease and a poor prognosis.

The mechanism of cutaneous metastasis remains elusive. Direct extension, hematogenous or lymphatic spread, spread along the ligaments of embryonic origin, and implantation of tumor cells may be involved.^[10]^ In addition, cutaneous metastases may also occur through lymphopenias spread, intravascular dissemination,^[11]^ direct extension of the tumor, surgical implantation, and spread along the urachus.^[12]^

The diagnosis of cutaneous metastasis is based on the morphologic appearance, histomorphology, and immunohistochemistry of the lesions, along with comparison with the primary tumor morphology if available.^[13]^ Cutaneous metastases have many clinical manifestations, such as a rapidly growing painless dermal or cutaneous nodules with intact overlying epidermis and lesions mimicking inflammatory dermatosis. Ulceration, bullae, nodules, and fibrotic processes are the most common presentations of cutaneous metastases.^[14]^

Considering the poor prognosis of cutaneous metastasis, treatment options include surgical excision, radiotherapy, chemotherapy, and targeted therapy. The treatment options mainly depend on the type and extent of the primary tumor and metastatic disease, along with the genetic characterization.^[19]^ Wide local excision of the cutaneous metastatic lesion is the preferred treatment for isolated lesions. For patients with multiple cutaneous metastases or unresectable lesions, systemic chemotherapy, targeted therapy, or even immunotherapy could be considered.^[20]^ Radiotherapy, isolated limb perfusion, polychemotherapy, interferon alpha injections, cryotherapy, laser ablation, radiofrequency ablation, imiquimod 5% cream, and oncogene-targeted therapy have also been examined.^[7]^

Previous studies have shown that BRAF mutation is a poor prognostic factor in colorectal cancer patients. BRAF-mutated colorectal cancer exhibits distinct clinicopathological features from wild-type BRAF cancer independent of the microsatellite instability status. Further, the optimal treatment for skin metastases with BRAF mutations needs to be further explored. Anti-VEGF therapy and chemotherapy are less effective in patients with BRAF mutations. BRAF V600 mutations are associated with rare objective responses to the BRAF inhibitor vemurafenib in patients with mCRC. Inhibition of BRAF by vemurafenib causes upregulation of EGFR, whose signaling activities can be impeded by cetuximab. A randomized trial of irinotecan and cetuximab with or without vemurafenib in BRAF-mutant metastatic colorectal cancer (SWOG 1406) showed that the addition of vemurafenib to the combination of cetuximab and irinotecan could result in a prolongation of progression-free survival (PFS) and a higher disease control rate, suggesting that simultaneous EGFR and BRAF inhibition is effective in BRAF V600-mutated CRC. PFS was improved with the addition of vemurafenib; these patients had a median PFS of 4.4 months, compared to 2.0 months in patients not receiving vemurafenib.^[16]^

However, palliative chemotherapy could be an ineffective treatment for these metastases. Schoenlaub et al reported that the median survival of patients with cutaneous metastasis of primary colorectal tumors was 4.4 months. Conversely, a retrospective study by Lookingbill et al^[17]^ showed a median survival of 18 months in patients with similar characteristics. Ueda et al^[11]^ reported a case of facial cutaneous metastasis of sigmoid colon adenocarcinoma with a postoperative survival of 37 months. Our patient responded well to chemotherapy and cetuximab with vemurafenib treatment and survived 6.5 months after presentation with skin metastasis. Although multiple lesions in the body had achieved partial remission, eventually, the patient developed bone metastasis. This outcome suggests heterogeneity of the tumor.

In the BEACON CRC study, the median overall survival time of the three-drug regimen encorafenib (ENCO)+ binimetinib (BINI)+ cetuximab was 9.0 months (95% confidence interval, 8.0–11.4).^[18]^ The combination of ENCO+ BINI+ cetuximab improved the OS and ORR in patients with BRAF V600E-mutant mCRC when compared with the current standard of care chemotherapy and had a safety profile consistent with the known safety profile of each agent.^[18]^ If this procedure could have been applied early in this patient to treat subcutaneous metastases, it might have changed the outcome.

## 4 Conclusion

In conclusion, cutaneous metastasis of colorectal cancer with BRAF V600E mutation is rare and should not be ignored. Cutaneous metastasis frequently indicates advanced disease and a poor prognosis in colorectal cancer. Early diagnosis, which requires careful physical examination, is key for effective treatment. Once changes in the skin exist, further examination should be implemented. More effective treatment modalities need further exploration.

## Author contributions

LLG, FP, QC, and SWY drafted the manuscript and revised it. LLG, QC, FP, and SWY participated in data collection. LLG, SGZ, and SWY collected cases and did the check. All authors read and approved the final manuscript.

## References

[1] Eom YH Kim EJ Chae BJ The distance between breast cancer and the skin is associated with axillary nodal metastasis. J Surg Oncol 2015;111:824–8. 25847102 10.1002/jso.23898

[2] Qi L Ding Y Analysis of metastasis associated signal regulatory network in colorectal cancer. Biochem Biophys Res Commun 2018;501:113–8. 29704502 10.1016/j.bbrc.2018.04.186

[3] Ahmed D Sunal P Sean K Cutaneous metastasis of rectal cancer: a case report and literature review. Perm J 2016;20:74–8. 10.7812/TPP/15-078 PMC4732799 26824966

[4] Taek-Gu Lee Sang-Jeon Lee Unusual case of solitary perineal subcutaneous metastasis from sigmoid colon cancer. Ann Coloproctol 2013;29:34–7. 23586014 10.3393/ac.2013.29.1.34 PMC3624980

[5] Plaza JA Perez-Montiel D Mayerson J Metastases to soft tissue: a review of 118 cases over a 30-year period. Cancer 2008;112:193–203. 18040999 10.1002/cncr.23151

[6] Kemal Y Odabaşi EA Kemal Ö Cutaneous metastasis of colon adenocarcinoma. Turk J Surg 2018;34:237–9. 30302429 10.5152/turkjsurg.2017.3298 PMC6173589

[7] Wang DY Ye F Lin JJ Cutaneous metastasis: a rare phenomenon of colorectal cancer. Ann Surg Treat Res 2017;93:277–80. 29184882 10.4174/astr.2017.93.5.277 PMC5694720

[8] Ha JY Oh EH Jung MK Choroidal and skin metastases from colorectal cancer. World J Gastroenterol 2016;22:9650–3. 27920486 10.3748/wjg.v22.i43.9650 PMC5116609

[9] Bittencourt MJS Imbiriba AA Oliveira OA Cutaneous metastasis of colorectal cancer. An Bras Dermatol 2018;93:884–6. 30484536 10.1590/abd1806-4841.20187610 PMC6256216

[10] Krathen RA Orengo IF Rosen T Cutaneous metastasis: a meta-analysis of data. South Med J 2003;96:164–7. 12630642 10.1097/01.SMJ.0000053676.73249.E5

[11] Shah SR Applebaum DS Potenziani S Cutaneous metastasis to the scalp as the primary presentation of colorectal adenocarcinoma. Dermatol Online J 2017;23 (11.): 29447644

[12] Komori K Kimura K Kinoshita T Cutaneous metastases from colorectal cancer: oncological behavior and surgical strategy. Am Surg 2016;82:e359–60. 28234169

[13] Hashimi Y Dholakia S Facial cutaneous metastasis of colorectal adenocarcinoma. BMJ Case Rep 2013;2013. 10.1136/bcr-2013-009875 PMC3822271 24177455

[14] Sheets N Powers J Richmond B Cutaneous metastasis of colon cancer: case report and literature review. W V Med J 2014;110:22–4. 24984402

[15] Lookingbill DP Spangler N Sexton FM Skin involvement as the presenting sign of internal carcinoma. A retrospective study of 7316 cancer patients. J Am Acad Dermatol 1990;22:19–26. 2298962 10.1016/0190-9622(90)70002-y

[16] Scott Kopetz Shannon L McDonough Randomized trial of irinotecan and cetuximab with or without vemurafenib in BRAF-mutant metastatic colorectal cancer (SWOG 1406). J Clin Oncol 2017;35: 4_suppl: 520–1520. 10.1200/JCO.20.01994 PMC8462593 33356422

[17] Lookingbill DP Spangler N Helm KF Cutaneous metastases in patients with metastatic carcinoma: a retrospective study of 4020 patients. J Am Acad Dermatol 1993;29 (2 Pt 1):228–36. 8335743 10.1016/0190-9622(93)70173-q

[18] Kopetz S BEACON CRC: a randomized, 3-Arm, phase 3 study of encorafenib and cetuximab with or without binimetinib vs. choice of either irinotecan or FOLFIRI plus cetuximab in BRAF V600E–mutant metastatic colorectal cancer. Ann Oncol 2019;30(4 suppl): mdz183.004, 10.1093/annonc/mdz183.004.

[19] Dong Z Qin C Zhang Q Penile metastasis of sigmoid colon carcinoma: a rare case report. BMC Urol 2015;15:20. 25887957 10.1186/s12894-015-0014-9 PMC4371621

[20] Fyrmpas G Barbetakis N Efstathiou A Cutaneous metastasis to the face from colon adenocarcinoma. Case report. Int Semin Surg Oncol 2006;3:2. 16457715 10.1186/1477-7800-3-2 PMC1368989

